# Preparing Aquatic Research for an Extreme Future: Call for Improved Definitions and Responsive, Multidisciplinary Approaches

**DOI:** 10.1093/biosci/biac020

**Published:** 2022-05-04

**Authors:** Lillian R Aoki, Margaret Mars Brisbin, Alexandria G Hounshell, Dustin W Kincaid, Erin I Larson, Brandon J Sansom, Arial J Shogren, Rachel S Smith, Jenna Sullivan-Stack

**Affiliations:** Department of Ecology and Evolutionary Biology, Cornell University, Ithaca, New York; University of Oregon's Data Science Initiative, Eugene, Oregon, United States; Woods Hole Oceanographic Institution, Woods Hole, Massachusetts, United States; Biological Sciences Department, Virginia Tech, Blacksburg, Virginia; National Oceanic and Atmospheric Administration, National Centers for Coastal Ocean Science, Silver Spring, Maryland, United States; Vermont EPSCoR and Gund Institute for Environment, University of Vermont, Burlington, Vermont, United States; Institute of Culture and Environment, Alaska Pacific University, Anchorage, Alaska, United States; Department of Geography, State University of New York University, Buffalo, Buffalo, New York; US Geological Survey's Columbia Environmental Research Center, Columbia, Missouri, United States; Department of Earth and Environmental Sciences, Michigan State University, East Lansing Michigan; Department of Biological Sciences, University of Alabama, Tuscaloosa Alabama, United States; Department of Environmental Sciences, University of Virginia, Charlottesville, Virginia, United States; Department of Integrative Biology, Oregon State University, Corvallis, Oregon, United States

**Keywords:** extreme event, long-term ecological research, marine, freshwater, climate change

## Abstract

Extreme events have increased in frequency globally, with a simultaneous surge in scientific interest about their ecological responses, particularly in sensitive freshwater, coastal, and marine ecosystems. We synthesized observational studies of extreme events in these aquatic ecosystems, finding that many studies do not use consistent definitions of extreme events. Furthermore, many studies do not capture ecological responses across the full spatial scale of the events. In contrast, sampling often extends across longer temporal scales than the event itself, highlighting the usefulness of long-term monitoring. Many ecological studies of extreme events measure biological responses but exclude chemical and physical responses, underscoring the need for integrative and multidisciplinary approaches. To advance extreme event research, we suggest prioritizing pre- and postevent data collection, including leveraging long-term monitoring; making intersite and cross-scale comparisons; adopting novel empirical and statistical approaches; and developing funding streams to support flexible and responsive data collection.

The increasing frequency and intensity of extreme  events driven by anthropogenic climate change pose a significant threat to human society and ecosystems alike (Li and Chakraborty 2020). Broadly, extreme events cause significant damage to infrastructure and loss to human communities. For example, between 2000 and 2019, nearly twice as many major natural disasters occurred around the world as did in the previous 20-year period, resulting in US$2.97 trillion in economic losses (UNDRR 2020). In addition to this hefty price tag, extreme events substantially alter ecosystems, often with dramatic consequences for biodiversity and ecosystem structure and function (Kendrick et al. 2019). Loss of structure and function can negatively affect ecosystems services and further exacerbate the vulnerability of ecosystems and the built environment to future extreme events. Understanding and predicting extreme event drivers, dynamics, and responses has emerged as an increasingly prominent research direction across natural and social sciences over the last few decades, demonstrated by the rapid growth of published research articles (figure 1). However, progress is hindered by unclear definitions of *extreme event* among and within disciplines and event types and by conflation of the cause of the extreme event with responses to the event (McPhillips et al. 2018). The resulting lack of clarity impedes research on extreme events and on the responses of socioecological systems, with negative consequences for communication, policy, and management.

**Figure 1.**
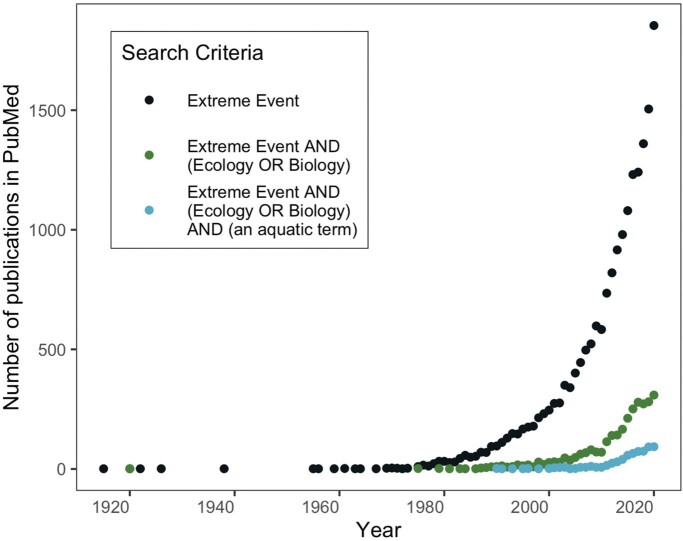
Trends in the annual number of published research articles on extreme events. The numbers of research articles published per year based on a PubMed search for articles including the term *extreme event* are shown in black, the numbers of articles per year resulting from a search including search terms *extreme event* and *biology* or *ecology* are shown in green, the number of articles per year resulting from a search including search terms *extreme event* and *biology* or *ecology* and an aquatic search term (*marine*, *aquatic*, *ocean*, *lake*, *stream*, *river*, or *coastal*) are shown in blue. The last year of data depicted in the graph is 2020.

Aquatic ecosystems and their provided services are disproportionately affected by extreme events that rapidly disrupt the hydrologic cycle (UNDRR 2020). Within this synthesis, we define *aquatic ecosystems* as including any freshwater (lakes, streams, wetlands), transitional (estuaries and coasts), and marine (intertidal and subtidal zones, open ocean) ecosystems (this usage is in keeping with the Association for the Sciences of Limnology and Oceanography, which defines aquatic science as “the study of the planet's oceanic and freshwater environments”; www-aslo-org/what-is-aquatic-science). Examples of extreme events that affect aquatic ecosystems include changes to the hydrologic regimes in river networks (i.e., more extreme high- and low-flow conditions) that significantly alter riparian plant communities, decrease streambank resilience, and degrade biodiversity (Tonkin et al. 2018). In the open ocean, marine heatwaves have become more frequent, intense, extensive, and persistent over the past decades, with direct impacts on marine species assembly, ecosystem stability, and fisheries (Frölicher et al. 2018). Although ecological responses are often ecosystem and event specific, the increasing threat of extreme events for aquatic ecosystems is likely to negatively affect both ecologically and societally important ecosystem services (e.g., water availability and biodiversity; Vörösmarty et al. 2010).

The context of what determines an “extreme” event is nontrivial. For example, questions of event extent (i.e., How large a geographic area did the event affect? How long did the event last?) and baseline or reference periods (i.e., What were the conditions before the event?) affect statistical thresholds such as the 90th percentile used to define the extreme nature of the event (Seneviratne et al. 2012). Still, extreme events should ultimately be delineated by the preevent context (e.g., by historical occurrence or exceedance benchmarks), enabling assessments against a baseline or reference period (Abrahams et al. 2013). Clear definitions enable comparisons between ecosystems; for example, climatological definitions of marine heatwaves, proposed by Hobday and colleagues (2016), enabled a global meta-analysis of marine heatwave responses across biological taxa and the identification of vulnerable regions in coming decades (Smale et al. 2019). Similar definitions have been applied to lakes to show patterns of increased length and intensity of lake heatwaves in the twenty-first century, with consequences for understanding lake biodiversity and ecosystem services (Woolway et al. 2021a). Formalized definitions may also enhance site-specific studies; for instance, in an analysis of 35 tropical cyclones that occurred in a North Carolina (in the United States) estuary over two decades, water quality and biogeochemistry experienced a regime shift caused by storms that exceeded 90th percentile thresholds for wind or precipitation (Paerl et al. 2018). The ability to make spatial and temporal comparisons places discrete events and ecological responses into context, increasing our understanding of phenomena such as species’ adaptation and acclimation to climate change (Duarte et al. 2018), ecosystem resilience (Cavanaugh et al. 2019), and vulnerability to regime shifts (Paerl et al. 2019).

Despite the susceptibility of aquatic ecosystems to extreme events and their important implications for both biodiversity and human well-being, the majority of studies on the ecological responses to these events have occurred in terrestrial ecosystems (Maxwell et al. 2019), with relatively poor understanding of responses across aquatic ecosystems. As researchers work to address this knowledge gap, we need to critically assess the methods and practices that ecologists employ to study extreme events in aquatic ecosystems. Understanding the status of current extreme event research will enable aquatic ecologists to improve research approaches, address gaps in understanding, and advance the management of extreme event responses in these critical ecosystems.

In the present article, we review and synthesize ecological studies on the effects of extreme events in aquatic ecosystems to determine how, where, and when ecologists study these events. Following this literature review, we identified several key issues in ecological research approaches to extreme events, including needs for clarity in definitions; improved strategies to capture spatial and temporal variation; further training for ecologists and multidisciplinary collaborators to successfully study extreme event responses; and suggestions for tools needed to conduct this research in the future. Below, we explore each of these topics in depth and offer suggestions to advance studies of extreme events across aquatic ecosystems.

## Synthesis of extreme events in aquatic ecosystems

To examine how ecologists study the effects of extreme events in aquatic ecosystems, we reviewed the scientific literature using the Web of Science Core Collection. Our search included all peer-reviewed English-language journal articles available from Web of Science and published from 1 January 1965 to 15 February 2019. We searched using the terms shown in supplemental table S1 to target three categories of ecosystems (freshwater, coastal, and marine) and four types of extreme events (heatwaves, storms, floods, and drought). We targeted these events because they are the most commonly described extreme events in the rapidly expanding climate attribution literature (Carbon Brief 2021); however, we were not exclusively interested in these event types. Some papers described other types of extreme events (e.g., an extreme cold spell; Boucek and Rehage 2014). To investigate a broader range of the extreme event literature, we included these papers, provided they met the other inclusion criteria by including observations of ecological responses to an extreme event occurring in an aquatic ecosystem. We excluded papers if they did not include observations of an extreme event but rather used extrapolation or modeling to predict the effects of a hypothetical extreme event, were review papers that discussed extreme events, were manipulative experimental work, or were focused on terrestrial ecosystems or paleoclimatology. We focused on observations of extreme events to understand how ecologists research these events and their ecological responses *in situ* and to explore challenges with observational approaches, such as capturing spatial and temporal variation. Although manipulative studies provide an alternative and important avenue to test and predict mechanisms of ecological response, these types of studies present a different set of research challenges and are beyond the scope of this review. Overall, the search yielded 215 unique papers, 49 of which we included in the review, after excluding papers that did not meet the criteria described above.

**Figure 2.**
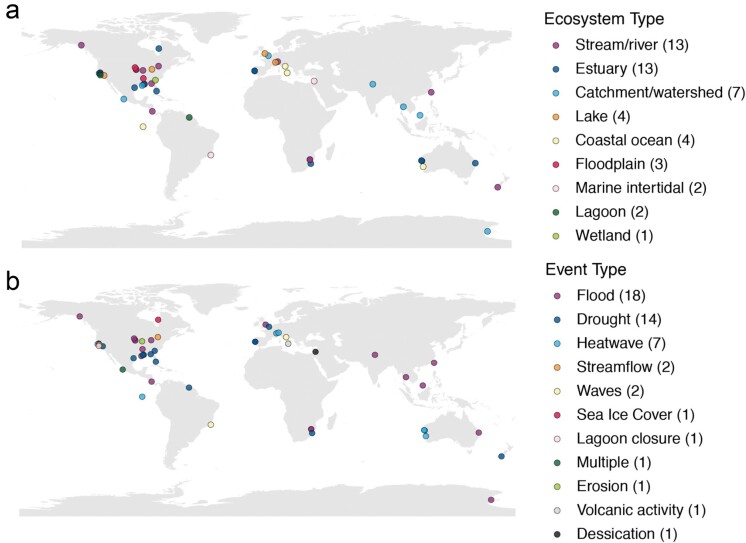
Studies included in the literature review covered a range of ecosystems (a) and event types (b). Ecosystem type and event type are ordered by frequency on the basis of the literature review and the number of studies for each type are included in parentheses in the figure legend. Event types refer to the proximate events (i.e., the specific physical conditions described as extreme by authors of reviewed manuscripts). Proximate events were often caused by or part of a distal event described by authors. For example, extreme wave energy (proximate event) described in a study was due to a tropical cyclone (distal event). A dynamic dashboard detailing additional characteristics of the reviewed papers is available at https://public.tableau.com/app/profile/margaret.mars.brisbin/viz/ExtremeEventsinEcologicalResearch/extreme_db.

We extracted relevant information from each study, including characteristics of the ecosystem, the extreme event (including definitions provided by the authors), the sampling design, and response variables (see Aoki et al. 2022 for the published data set). Although the literature review captured studies from all continents and 21 countries, the majority of studies were of ecosystems in North America and Europe (figure 2). Geographic bias is a known limitation within the English-language ecological literature, which may create bias in scientific knowledge and applications (e.g., in biodiversity policies; Di Marco et al. 2017, Culumber et al. 2019). Despite these limitations, we believe this snapshot of aquatic research on extreme events provides a solid foundation for assessing approaches to extreme event research.

In comparing events across papers, we quickly encountered the challenge of disentangling proximate and distal extreme events. On the basis of descriptions by the authors, we identified proximate events as the immediate source of extreme conditions, such as a flood causing high water levels. The flood may have been caused by a storm or weather system, which we identified as the distal extreme event that created the conditions for the proximate event, following the descriptions provided by the authors. This distinction was relevant because the same type of event could be either distal or proximate depending on the study; for example, Boersma and colleagues (2016) studied the response of invertebrate communities to extreme drought in mountain lakes, whereas Osterback and colleagues (2018) studied the response of salmon and steelhead to lagoon closure, caused by a prolonged drought. In other papers, no distinct phenomenon was identified as a distal event. For simplicity, we chose to compare proximate event types, and across the 49 papers included in the review, we identified 10 proximate event types occurring in nine ecosystems (figure 2). Storms were consistently described as distal events, causing other physical conditions such as floods or waves that were described as the proximate extreme events.

## Defining extreme events in aquatic ecosystem research

**Figure 3.**
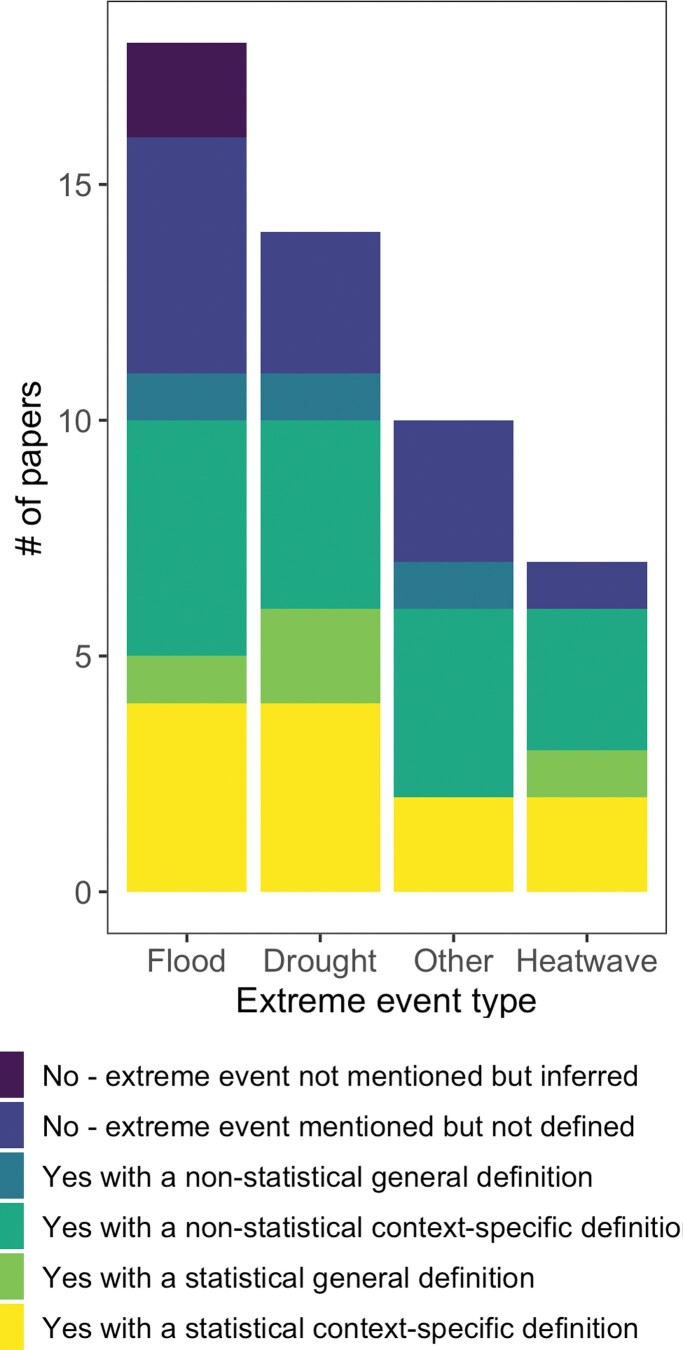
Use of definitions of *extreme event* in studies included in the literature review. Studies included freshwater, marine, and coastal ecosystems; event types included floods, drought, heatwave (the three most studied event types), and other (streamflow, waves, sea ice cover, lagoon closure, erosion, volcanic activity, desiccation, and multiple). A total of 49 studies, published from 1997 to 2018, were included in the review.

In our review, studies of extreme events in aquatic ecosystems often lacked an explicit definition of *extreme*. Approximately one-third of the 49 papers used a statistical definition (e.g., a definition based on a return interval or a metric of variation from mean conditions); one-third used a general, nonstatistical definition (e.g., an absolute increase in temperature of 2–4 degrees Celsius described as atypical or unusual); and one-third gave no definition (figure 3). These results align with similar analyses in terrestrial ecosystems and underscore a lack of consistency regarding what is considered an “extreme” event in ecology. For example, a recent review that included 60 ecological studies of extreme events found that roughly half explicitly defined an extreme event, whereas the other half relied on implicit definitions (McPhillips et al. 2018). Another synthesis of 564 drought-related ecological studies found that only 32% provided any concrete definition (Slette et al. 2019). Clear definitions of what constitutes *extreme* are needed to improve our ability to generalize from site-specific events, including comparing findings through time and space.

More precise definitions of extreme events will improve the application of ecological research by facilitating communication between disciplines and multidisciplinary research. Extreme events are a research focus across disciplines (Broska et al. 2020), but explicit definitions are lacking across fields; a review covering the fields of climatology, earth sciences, ecology, engineering, hydrology, and social sciences showed that papers explicitly defined extreme events less than half the time in all fields (McPhillips et al. 2018). At the same time, there is greater recognition of the interconnectedness of socioecological systems and the interactions that can amplify risk from extreme events (Raymond et al. 2020), along with greater efforts to integrate ecological forecasting into policymaking (Dietze et al. 2018). Therefore, clear communication across fields is vital (Pennington et al. 2013, McPhillips et al. 2018). A practical example is the development of coral reef management plans that rely on specific metrics of degree-heating weeks to delineate extreme thermal stress and to identify appropriate management strategies (Beeden et al. 2012, Darling et al. 2019). Adopting explicit definitions of extreme events is a key step for ecologists to improve their contributions to socioecological understanding, adaptation, and resilience.

Adopting a universal definition of *extreme* is difficult in an era of accelerating environmental change. Trends in climate will influence the magnitude of extreme climatic and weather events; for example, rising temperatures will increase rainfall and runoff extremes (Yin et al. 2018). Temporal records of disturbance events may therefore exhibit nonstationarity (i.e., changes in mean or variance over time; Betancourt 2012, Slater et al. 2021). However, these trends highlight the importance of avoiding implicit or intuitive definitions (Salt 1979). Rather than suggest a standard definition of *extreme* across all aquatic ecosystems and event types, we recommend that ecologists identify and justify the definition of *extreme* applied in a given study. Ecologists should also pay close attention to their definitions of event types, including distinguishing proximate and distal events and identifying compound events when appropriate. By identifying specific physical observations, such as temperature, precipitation, or wind, as the proximate event, ecologists can more closely link observed ecological responses to potential drivers within the context of a distal event, such as a storm. Compound extreme events that include multiple physical drivers are of increasing concern for socioecological systems (Zscheischler et al. 2018). By specifying the metrics of interest in a study and identifying the broader context, ecologists can improve comparisons across event types and ecosystems.

We propose including three elements in any definition of extreme events: a general definition of the type of event, such as a flood, heatwave, or drought; appropriate metrics used to evaluate the conditions of the event, such as temperature during a heatwave; and an explanation of when the selected metric signifies extreme conditions, such as exceeding the 95th percentile. We give examples of this approach in table 1. These examples are not comprehensive; hydrological and climatic events are dynamic and complex, and many metrics and indices are used in the scientific literature. Instead, they demonstrate how the elements of a complete definition reduce ambiguity. Comprehensive categorization schemes help account for different spatiotemporal dimensions of extreme events, such as the duration, intensity, frequency, and magnitude, and can provide more detailed information to support inferences (Hobday et al. 2018). Furthermore, precise definitions of extreme events can differentiate research questions that investigate the ecological responses to a climate extreme as opposed to the climatic conditions that produce an extreme ecological response. As an example of these contrasting approaches, ecologists may study changes in primary productivity in response to extreme drought or, alternatively, study extreme mortality in response to temperatures that do not cross a statistical threshold (van de Pol et al. 2017).

**Table 1.** Representative examples of complete definitions of *extreme events* in aquatic ecosystems, including a general definition of the type of event, appropriate metrics to evaluate specific conditions of the event, and thresholds or other measures of the extremity of the metrics.

Event type	General definition	Metrics of specific conditions	What is extreme?
Storm	Weather events “associated with heavy precipitation, strong wind, and the passage of warmer or cooler air masses” (Stockwell et al. 2020)	Precipitation, wind speed, or water flow, compared with relative or absolute thresholds.	A relative threshold for extreme precipitation is exceeding the 95th percentile. An absolute threshold for extreme precipitation is 50.8 millimeters per day in the United States and 100 millimeters per day in China (Seneviratne et al. 2012) 90th percentile of maximum hourly wind speed and 90th percentile of maximum weekly water flow have also been used as extreme storm thresholds (Paerl et al. 2019)
Drought	“A period of abnormally dry weather long enough to cause a serious hydrological imbalance” (Field et al. 2012)	Annual precipitation Palmer Drought Severity Index, PDSI (Palmer 1965)	Annual precipitation below the 10th percentile (Breshears et al. 2005) PDSI less than –3 (e.g., (Zhang et al. 2019)
Flood	A “temporary covering of land by water outside its normal confines” (Rojas et al. 2013)	Return time or return period (interval of time between occurrences of a flood)	Less than 1-in-100 year probability, indicating the discharge that has a probability of being exceeded in a given year of 0.01 (Milly et al. 2002)
Heatwave	“A discrete, prolonged, anomalously warm water event in a particular location” (Hobday et al. 2016)	Temperature, compared with relative or absolute thresholds	For marine heatwaves, a period of at least 5 consecutive days when water temperatures exceed the 90th percentile of local, long-term climatology (Hobday et al. 2016). Extreme heatwaves occur when temperatures exceed the 90th percentile threshold by more than four times the difference between mean and 90th percentile temperatures. (Hobday et al. 2018).

## Spatiotemporal considerations for capturing aquatic extreme events

Extreme events are often sporadic in time and variable across space, which creates a challenge for researchers seeking not only to demarcate the event itself but to measure the ecological responses to such intense disturbances (Redmond et al. 2019). With limited monitoring resources, trade-offs in spatial and temporal resolution often lead to high temporal frequency observations at low spatial resolution or high spatial resolution monitoring at low temporal frequency (Krause et al. 2015). Mismatches between the scale of the study and of the event can be accentuated by difficulties associated with sampling extreme events that occur across large geographies (e.g., drought, hurricanes, marine heatwaves) or in remote areas without established sampling infrastructure. These limitations can prevent capture of the ecological responses to extreme events. Our literature analysis provided an opportunity to evaluate these potential mismatches and to identify strategies to improve spatiotemporal considerations.

To compare the spatial range of an extreme event with that of a study, we examined two metrics: the spatial scale of the full study (i.e., the range encompassing all sampling units) and the spatial scale of individual sampling units. Several papers used different spatial scales for multiple response variables within the study, leading to 53 comparisons between extreme event scale and study spatial scale for the 49 papers. For approximately half of these comparisons, the spatial scale of the full study captured data at the same spatial scale as the extreme event itself (55%; 29 of 53 spatial comparisons; see supplemental figure S1). For example, two studies at a large spatial scale (more than 1000 square meters [m^2^]) used remotely sensed data to measure watershed-level responses (more than 1000 square meters) to extreme floods (Trigg et al. 2013, Chauhan et al. 2017).

Few studies (13%; 7 of 53 spatial comparisons) matched the spatial scale of the sampling unit with the event itself; instead, researchers generally sampled at smaller spatial scales for large events, which likely corresponded to the most relevant sampling scale for the response variable of interest (supplemental figure S2). For example, a study included in our review took soil samples from ninety (less than 1 m^2^) sampling points along transects spread across 1000 m^2^ of a creek watershed to measure faunal and nutrient responses to extreme floods (Nielsen et al. 2012). In this case, the scale of the study matched the scale of the event while using a smaller scale sampling unit. Capturing the full spatial scale of extreme events is challenging but critical to understanding ecological responses.

To compare the temporal scale of an extreme event with that of a study, we considered how long each event lasted versus how long the investigators monitored the ecological responses. We found that data are often collected at longer time frames (e.g., years) relative to the event (e.g., months; supplemental figures S3–S5). This pattern likely indicates the important role of long-term research programs—for example, the Long-Term Ecological Research (LTER) Network, the National Ecological Observatory Network (NEON), the National Estuarine Research Reserve (NERR)—in capturing extreme events (Gaiser et al. 2020). However, these long-term monitoring programs are often not designed to capture extreme events; for example, weekly or monthly sampling could miss short-term algal blooms, or concentrated summer sampling could miss rare spring heatwaves. The utility of extreme event research can be enhanced by employing targeted short-term studies and regional surveys implemented rapidly during developing extreme events (Redmond et al. 2019). Another temporal challenge, especially for opportunistic studies, is the lack of data measured consistently before, during, and after extreme events. In our review, 49% (26 of 53 temporal comparisons) of studies collected data at all three of these time points, which is ideal for assessing the response to extreme events. However, 13% (7 of 53 temporal comparisons) of studies collected data only during (4 temporal comparisons) or only after (3 temporal comparisons) an extreme event, limiting the ability to assess change in response to an event. The remaining studies (33 of 53) collected data at two time points (before and during, during and after, before and after). Therefore, we recommend collecting data from at least two time points to best evaluate the effects of an extreme event.

Overall, the comparisons between spatial and temporal scales of extreme events and ecological studies highlight the challenges of capturing heterogeneous and unexpected disturbance in dynamic aquatic environments. Moreover, individual extreme events are difficult to predict, and trends of increasing frequency and severity emphasize the need for researcher preparedness (Jones et al. 2016). Spatial scale is often inherent to addressing scientific questions and developing methodologies; identifying appropriate sampling plans and tools is a challenge for capturing spatial variation in ecological responses, particularly in opportunistic studies of extreme events. Similarly, temporal scales are often constrained by ongoing long-term monitoring programs, short-term studies, or safety or logistic concerns during an extreme event. To address these challenges, we recommend developing contingency plans and protocols that can be executed rapidly during developing extreme events (Redmond et al. 2019). For example, to account for spatial heterogeneity, ecologists can pair remotely sensed technologies that integrate across larger areas with targeted *in situ* sampling or leverage distributed sampling efforts through collaboration. Likewise, autonomous sampling devices, such as remotely deployed cameras to monitor meso- and phytoplankton communities (Anglès et al. 2015, Grossmann et al. 2015), can alleviate safety and logistic concerns associated with sampling during extreme events. Although these approaches have trade-offs (e.g., substantial upfront or installation costs, periodic maintenance and retrieval of autonomous devices, data sets that require cyberinfrastructure and expertise to house and process the data), multidisciplinary teams, distributed networks, and long-term monitoring can help to develop and implement sampling protocols that better capture extreme events and their responses across spatiotemporal scales.

## Transcending disciplinary boundaries

**Figure 4.**
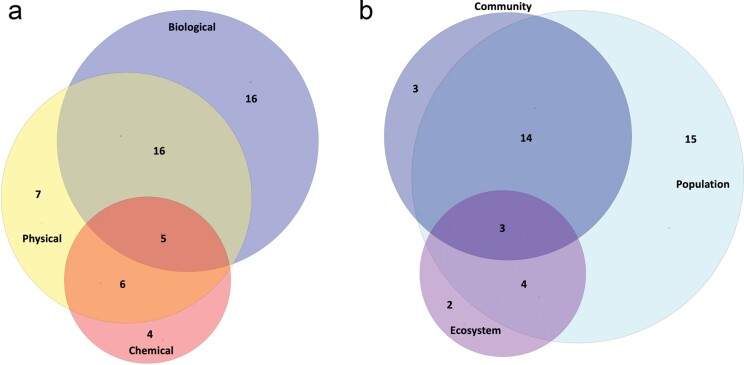
Venn diagrams of the number of studies measuring biological, physical, and chemical response variables (a) and for the studies measuring biological variables, the number of studies measuring different levels of ecological organization (b). The numerals reflect the number of studies measuring each variable type; the numerals in overlapping areas indicate studies measuring two (or three) variable types. The Venn diagrams are scaled such that the size of each circle corresponds to the total number of studies that included that variable and the area of overlap reflects the number of studies including one or more variables.

A holistic understanding of the effects of extreme events on aquatic ecosystems requires that ecologists engage multiple disciplines. Broadly, ecological research requires integrating biological, chemical, and physical responses; this multidisciplinary integration is even more important to understand the ecosystem-wide responses to extreme events, but our review suggests limited integration. Of the 49 studies we examined, 88% measured a biological response variable, whereas only 10% measured biological, physical, and chemical response variables concurrently (figure 4a). Of the studies that measured a biological response variable, 73% looked at population-level responses (e.g., density, abundance), 45% looked at community-level responses (e.g., species richness, food web structure), and 22% looked at ecosystem-level responses (e.g., enhanced vegetation index, biomass; figure 4b). Fifty percent of studies that measured a biological response variable looked at two or more levels of biological organization (i.e., population, community, ecosystem level), but only 7% of the studies measured population, community, and ecosystem-level biological responses (figure 4b). We acknowledge that we focused our review on a small number of studies and that ecologists may measure variables that are not reported in the primary literature. Regardless, the narrow focus of extreme event studies on specific types of ecological responses presents an issue: Ecologists increasingly prioritize integrating across biological scales, and understanding how changes at one level of organization (e.g., population size) may result in changes at higher levels of organization (e.g., ecosystem metabolism; Heffernan et al. 2014, Rüegg et al. 2021). Thoroughly understanding ecological links to the physical and chemical environment and relationships of responses across scales will require more collaboration within and across disciplines.

Over 70% of the studies we reviewed explicitly framed their research in terms of climate change or linked measured responses to climate change. As such, we recommend collaboration between ecologists, hydrologists, and climatologists. For example, the 2013 floods in Colorado, in the United States (Larson et al. 2018, Poff et al. 2018) were an extreme event probabilistically, representing a 1-in-50 year to 1-in-500 year flood, depending on stream location (Gochis et al. 2015). However, the event did not have a climate change signature, a claim based on downscaled climate simulations that showed that the event was neither caused by greenhouse gas forcing or sea surface temperature forcing (Herring et al. 2014). Therefore, although studies of the 2013 floods identified ecological responses to extreme events made more likely under climate change, they did not explicitly measure the responses to climate change. Therefore, underlying drivers of climate change may have been missed. Rather, ecologists should work with climatologists to better predict future changes and identify which extreme events will have increased or decreased frequencies under current and future climate change predictions. If one impetus for studying extreme events in ecology is to better predict future changes, we also need to understand which extreme events are more or less likely under climate change, to properly contextualize ecological studies of extreme events. Attribution science (causally linking extreme events to climate change) has developed rapidly over the past decade (Bellprat et al. 2019), but ecologists may not always have direct evidence that a specific extreme event is in fact attributable to climate change. More collaboration with climate scientists would help bridge this gap. Climate science is already a multidisciplinary field; additional collaboration between climate scientists, meteorologists, hydrologists, and ecologists would integrate these often disparate bodies of knowledge in aquatic ecosystems.

Taking collaboration a step further, more convergence science—in the present article, used to describe problem-driven science that transcends disciplines and existing scientific boundaries—is needed to connect climate change to extreme events and, subsequently, to changes in ecology and effects on humans and infrastructure (Grimm et al. 2000, Alberti et al. 2020, Des Roches et al. 2021). This type of science can involve scientists from many disciplines (e.g., ecologists, climatologists) coming together to tackle complex problems. Many of the studies we reviewed focused on floods or droughts, which are commonly observed extreme events that have consequences for human infrastructure (e.g., wastewater, Kohler et al. 2020; road and bridges, Setunge et al. 2014; agricultural systems, Mcleman and Smit 2006). Collaborations between natural and social scientists and engineers, such as through the National Socio-Environmental Synthesis Center, urban LTER sites (Baltimore Ecosystem Study, Central Arizona-Phoenix, Minneapolis-St. Paul), and long-term agricultural research sites, can construct new paradigms for understanding disturbance ecology in socioecological systems (Grimm et al. 2000, Wright Morton et al. 2015, Grimm et al. 2017, Gaiser et al. 2020). An illustrative series of case studies is from the Baltimore LTER site, where heavy rains and flooding from hurricanes and cyclones cause millions of dollars in residential building damage (Li et al. 2020). Long-term data from the LTER site supported an analysis of how urbanization influenced flooding following a record rainfall event (a 100–300-year return interval) caused by an extratropical cyclone in 2004 (Ntelekos et al. 2008). Rainfall during this storm was influenced by the friction effects of canopy cover, changing wind speed and direction, which subsequently altered storm movement in the Baltimore metropolitan area (Ntelekos et al. 2008). Peak discharges in urban streams were regulated by the stormwater management systems in place in each catchment (Meierdiercks et al. 2010). Nearly a decade later, in October 2012, the US Atlantic Coast was battered by Superstorm Sandy. The long-term data available before and after the event at the Baltimore LTER site revealed that ecosystem metabolism in these urban streams was resilient to extreme flooding (Reisinger et al. 2017). This study suggests an interaction among the built environment, synoptic-scale systems, and ecological processes that mediates responses to extreme events. These insights are an example of the emergent feedback loops among ecological, socioecological, and meteorological systems and underscore the need for work that transcends traditional academic disciplines to fully reveal the drivers of extreme events in aquatic ecosystems.

The need for multidisciplinary science to study extreme events highlights the need for enhanced training for ecological researchers. Research teams may include scientists across the fields of climatology and meteorology, engineering, sociology, city and regional planning, economics, and beyond. Although ecology is inherently interdisciplinary (Reyers et al. 2010, Weathers et al. 2016), training focused specifically on conducting research in large, collaborative, and multidisciplinary teams will enable ecologists to work effectively across disciplines, institutions, and ecosystem boundaries (Cheruvelil et al. 2014, Cheruvelil and Soranno 2018, Farrell et al. 2021). Following Cheruvelil and Soranno (2018), we suggest that by expanding ecologists’ skill sets and collaborations using the framework of team science, we can advance the multidisciplinary research of extreme events.

## Recommended approaches to enhance studies of extreme events

As research on extreme events accelerates, we recommend ecologists and their collaborators leverage these specific tools and approaches to address research challenges: collecting pre- and postevent data at specific monitoring sites, collecting data that enables intersite and cross-site comparison, adopting novel empirical and statistical approaches, and increasing available funding sources for immediate study of extreme events.

### Collecting pre- and postevent data at specific monitoring sites

One challenge of assessing ecosystem responses to extreme events is sufficient data availability before, during, and after an event to contextualize a response. Given adequate time prior to the event (e.g., prior to a developing hurricane), sampling can collect crucial data. This opportunistic approach requires that resources (e.g., money and labor) are available in a timely fashion. However, these resources are not always readily available. Furthermore, failing to collect enough preevent data to capture inherent temporal variability in a response variable can limit the ability to draw conclusions about an event's impact. As such, long-term baseline data of chemical, biological, and physical parameters in aquatic ecosystems are inherently valuable but require dedicated infrastructure and funding for data collection, storage, and synthesis (Hampton et al. 2013, Lohner and Dixon 2013, Huang et al. 2020).

In the United States, several organizations carry out long-term ecological monitoring at distributed sites across targeted biomes, including the LTER Network, NEON, NERR, as well as Long-Term Research in Environmental Biology sites, the US Geological Survey (USGS) hydrologic monitoring stations, and the US National Park Service, which include sites or stations that are distributed throughout the United States and its associated territories (Fancy et al. 2009). International programs, such as the International LTER, the Global Lakes Ecological Observatory Network (Klug et al. 2012), the French Critical Zone Observatory (CZO) stations, and the Organization of Biological Field Stations enable similar data collection approaches globally (Tydecks et al. 2016). Together, these monitoring programs capture spatiotemporal characteristics at specific sites distributed across targeted biomes, ecoregions, and critical zones (e.g., Peters et al. 2014, Brantley et al. 2017, Gaiser et al. 2020), and through site-specific and network-level studies that facilitate collaboration and multidisciplinary advancements (e.g., Haberl et al. 2006, Johnson et al. 2010). Although the goals of each program may vary, the primary objective across these organizations is to provide a unified platform for understanding a diverse array of ecosystems spanning multiple spatial and temporal scales and to create legacies of well-designed and documented observations, experiments, and data archives.

The sites in these networks have already enabled critical study of site-specific responses to extreme events. When long-term data are available, the response to an extreme event can be evaluated rigorously at the site level. For example, both the Florida Coastal Everglades and Virginia Coastal Reserve US LTER sites have used their long-term data to understand the effects of strong tropical storms on coastal ecosystems (Gaiser et al. 2020), and US CZO sites have used their baseline data to understand response of stream water quality to wildfire (Brantley et al. 2017). In another instance, researchers combined data from existing long-term infrastructure and targeted data collected immediately after Hurricane Harvey hit the coasts of Texas and Louisiana (in the United States) in August 2017 to understand coastal responses to the hurricane (Patrick et al. 2020).

Given that many extreme events are becoming more frequent, we encourage such programs to evaluate how well their monitoring programs might capture the ecological responses of potential extreme events. For example, monitoring programs should assess whether sampling protocols sufficiently target species resistance and resilience traits to provide or enhance a mechanistic and predictive understanding of likely event responses (Leigh et al. 2015). Where existing experimental designs might fail to replicate the extreme and variable conditions of an event or fail to capture important thresholds, programs might consider adapting their designs—for example, by implementing a regression or gradient approach (Cottingham et al. 2005, Kreyling et al. 2014).

### Collecting data that allows intersite and cross-scale comparison

As was noted above, several efforts exist to collect detailed ecological data at target sites (e.g., LTER, NEON, USGS). In addition to addressing site-specific responses to extreme events, a coordinated network or distribution of sites that capture a diverse array of ecosystems can help facilitate comparison and synthesis by enabling both intersite and cross-scale studies of how specific populations, communities, and ecosystems are affected by extreme events. Standardized long-term data provided by research and monitoring networks can also enable robust comparison and synthesis of patterns and responses across scales (Heffernan et al. 2014). For example, an existing network of *in situ*, automated sensors in lakes and reservoirs in northeastern North America allowed researchers to evaluate how watershed-lake characteristics modulated abiotic and biotic responses to a single tropical cyclone (Klug et al. 2012). Such coordinated efforts may enable regional, continental, and even global studies (Peters et al. 2014, Brantley et al. 2017) that transcend traditional site-level understanding of the effects of extreme events on various ecosystems. Insight gleaned from these intersite and cross-scale comparisons better enable us to predict ecological responses to extreme drivers of change and more effectively manage these ecosystems. These approaches could be augmented by the use and development of centralized data repositories, such as the LTER's Environmental Data Initiative (https://environmentaldatainitiative.org/edi), DataONE (www.dataone.org), and the CZO hub at CUAHSI (https://criticalzone.org/learn-more.html).

### Adopting of novel empirical and statistical approaches

The adoption of novel empirical tools can enhance the ability of monitoring programs and long-term research studies to capture the effects of extreme events. For example, remotely sensed data, including unmanned arial vehicle, LIDAR (for *light detection and ranging*), and satellite imagery, have proven useful in documenting both aquatic and marine responses to extreme events (Wang et al. 2010). Remote sensing has detected changes in river geomorphology after an extreme flood (Tamminga et al. 2015), shifts in coral reef structure after an El Niño event (Ben-Romdhane et al. 2018), and changes in coastal wetland vegetation structure in response to major floods (Tahsin et al. 2018). Similarly, automated sensors capture data when researchers cannot measure safely or logistically in person, such as river floods (Burns et al. 2019) or tsunamis (Blain et al. 2004). High-frequency sensor technology has identified the role of sporadic storm pulses in disproportionately driving nutrient and carbon exports in rivers, which are challenging to capture using conventional grab-sampling approaches (Hartmann et al. 2014, Burns et al. 2019, Godsey et al. 2019). As another example, the use of open-access, low-cost sensors, particularly when paired with active community engagement (citizen science; Mao et al. 2019, Njue et al. 2019), has been instrumental in expanding watershed monitoring programs (e.g., Ensign et al. 2019) and filling data gaps when scientists themselves cannot visit a site postevent (Tauro et al. 2018, Guswa et al. 2020). In addition, the adoption of novel molecular biomonitoring approaches (e.g., environmental DNA; Thomsen and Willerslev 2015) and high-throughput *in situ* imaging (e.g., camera traps; for a review, see Farley et al. 2018) have more accurately recorded population and community shifts in response to major disturbance. These approaches have been successfully applied to collect data and assess the responses to extreme events across freshwater (e.g., Curtis et al. 2020, Mächler et al. 2021) and marine (Anglès et al. 2015, Grossmann et al. 2015, Ares et al. 2020, DiBattista et al. 2020) species that are challenging to identify or collect using conventional species-capture approaches. The empirical tools listed above, particularly when used in combination with conventional sampling approaches, can provide robust biological, chemical, and physical data to maximize the power and timeliness of ecosystem studies.

Furthermore, networked long-term records generate large, heterogeneous data sets (big data) that require novel applications of modern statistical approaches (e.g., Greig-Smith and Cragg 1964, Hampton et al. 2013, Durden et al. 2017, LaDeau et al. 2017). Although the appropriateness of any given statistical approach will vary on the basis of the scientific question at hand, recent advances in machine learning tools have enhanced our ability to understand responses to and predictions of extreme events (Rammer and Seidl 2019). For example, the application of automated neural networks and clustering analysis have facilitated the event-scale classification of riverine biogeochemical responses to extreme river flood events (e.g., Hamshaw et al. 2018, Javed et al. 2021), and biogeochemical responses to compound events in the ocean (Gruber et al. 2021). Other studies have focused on the utility of machine learning algorithms to improve capacity for forecasting future extreme events. For example, studies have applied random forests or regression trees to forecast hypoxia or high-temperature events in lakes (Politikos et al. 2021, Woolway et al. 2021b), used neutral networks to predict responses of estuaries and intertidal zones to changing sea level (e.g., Guillou and Chapalain 2021), and leveraged self-organizing maps to predict fish kills (Chen et al. 2020). Although our list of statistical tools is not exhaustive, we contend that the adoption of modern statistical approaches, especially those that can leverage heterogeneous data streams and multiple sources of uncertainty (Dietze 2017), will advance understanding of how aquatic and marine ecosystems, communities, and populations respond to extreme events.

### Increasing available funding sources for immediate study of extreme events

Responsive funding sources will be key to supporting the research needed to capture major disturbance events and their cascading ecological responses in freshwater, coastal, and marine ecosystems. Established long-term monitoring infrastructure, such as the networks mentioned above (e.g., LTER, NEON, USGS), will be critical to provide consistent and multidisciplinary data records to compare baseline conditions with extreme events. These networks are often place bound, with relatively immobile resources. LTER and NEON sites, for example, have long-term monitoring efforts in specific locations within a larger region or biome. However, some extreme events will occur in locations without an existing monitoring site, necessitating opportunistic sampling in addition to long-term monitoring. Such prompt implementation of strategic sampling will likely require nonconventional and adaptable approaches to funding and resource allocation. For example, in addition to NEON's core and gradient (previously referred to as *relocatable*) sites, researchers can request mobile, highly configurable sensor arrays, known as *mobile deployment platforms*, designed for rapid deployment to capture ecological events.

Some funding sources do exist to support responsive research. In the United States, the National Science Foundation (NSF)’s Rapid Response Research (RAPID) program uses a combination of shorter proposal length and a more expedient review process to disperse funds with urgency. Although funding sources such as the NSF-RAPID program facilitate quicker responses to major natural and anthropogenic events, they have several notable disadvantages: The duration of the grant is short, often less than 1 year, which does not allow the assessment of longer-term responses without additional funding; collaborations already need to be in place to apply for and take advantage of this funding source, potentially limiting the ability of multidisciplinary teams to mobilize in response to an event; and the process and procedure for proposal review and award can be lengthy (more than 6 months), despite immediate need for funds. Given these limitations, we urge funding agencies to design programs and resources capable of strategically supporting flexible and immediate data collection. Under climate change, extreme events will occur outside the bounds of previous experience and scientific infrastructure, including resource availability and collaboration. As such, funding sources and researchers will need to adapt to these new conditions and move beyond traditional approaches.

## Conclusions

Our review of the ecological literature on extreme events in aquatic ecosystems highlights specific ways to improve research approaches. First, we urge ecologists to better characterize and define the event they are studying. Clear identification of the metrics used to classify events and definitions of what constitutes *extreme* will improve our ability to generalize across events and ecosystems. Second, we encourage ecologists to develop sampling protocols that capture data at sufficient temporal and spatial resolution to contextualize events and their ecological responses in light of natural spatial and temporal variability. We further encourage collaboration across disciplines, leveraging intersite and long-term networks, and adoption of novel empirical and statistical tools to improve extreme event research. Finally, we call on funding agencies to balance support for long-term monitoring programs with improved funding and resources to support strategic, flexible, and rapid data collection. As the world experiences extreme events with increasing frequency and intensity, we hope this review and resulting recommendations will help the scientific community to develop a more comprehensive understanding of extreme events in aquatic ecosystems.

## Supplementary Material

biac020_Supplemental_File

## Author Biographical

At the time of writing, Lillian R. Aoki (lra53@cornell.edu) was affiliated with Department of Ecology and Evolutionary Biology at Cornell University, in Ithaca, New York; she is now affiliated with the University of Oregon's Data Science Initiative, in Eugene, Oregon, in the United States. Margaret Mars Brisbin is affiliated with the Biology and Marine Chemistry and Geochemistry Departments at the Woods Hole Oceanographic Institution, in Woods Hole, Massachusetts, in the United States. Alexandria G. Hounshell was affiliated with Biological Sciences Department atVirginia Tech, in Blacksburg, Virginia, and is now affiliated with the National Oceanic and Atmospheric Administration, National Centers for Coastal Ocean Science, in Silver Spring, Maryland, in the United States. Dustin W. Kincaid is affiliated with Vermont EPSCoR and Gund Institute for Environment, at the University of Vermont, in Burlington, Vermont, in the United States. Erin I. Larson is affiliated with Institute of Culture and Environment at Alaska Pacific University, in Anchorage, Alaska, in the United States. Brandon J. Sansom was affiliated with the Department of Geography at the State University of New York University at Buffalo, in Buffalo, New York, and is now affiliated with the US Geological Survey's Columbia Environmental Research Center, in Columbia, Missouri, in the United States. Arial J. Shogren was affiliated with the Department of Earth and Environmental Sciences at Michigan State University, in East Lansing Michigan, and is now affiliated with the Department of Biological Sciences at the University of Alabama, in Tuscaloosa Alabama, in the United States. Rachel S. Smith is affiliated with the Department of Environmental Sciences at the University of Virginia, in Charlottesville, Virginia, in the United States. Jenna Sullivan-Stack is affiliated with the Department of Integrative Biology at the Oregon State University, in Corvallis, Oregon, in the United States.

## References

[bib1] Abrahams C , et al. 2013. The Impact of Extreme Events on Freshwater Ecosystems. British Ecological Society.

[bib2] Alberti M , et al. 2020. The complexity of urban eco-evolutionary dynamics. BioScience 70: 772–793.

[bib3] Anglès S , Jordi A, Campbell L. 2015. Responses of the coastal phytoplankton community to tropical cyclones revealed by high-frequency imaging flow cytometry. Limnology and Oceanography 60: 1562–1576.

[bib4] Aoki LR , Brisbin MM, Hounshell AG, Kincaid DW, Larson E, Sansom BJ, Shogren AJ, Smith RS, Sullivan-Stack J. 2022. Review of Ecological Research Approaches for the Study of Extreme Events in Aquatic Ecosystems, ver. 1. Environmental Data Initiative. 10.6073/pasta/6cd8d60e00b384d718715c9882da74ae.

[bib5] Ares Á , Brisbin MM, Sato KN, Martín JP, Iinuma Y, Mitarai S. 2020. Extreme storms cause rapid but short-lived shifts in nearshore subtropical bacterial communities. Environmental Microbiology 22: 4571–4588. 33448616 10.1111/1462-2920.15178

[bib6] Beeden R , Maynard JA, Marshall PA, Heron SF, Willis BL. 2012. A framework for responding to coral disease outbreaks that facilitates adaptive management. Environmental Management 49: 1–13. 22042407 10.1007/s00267-011-9770-9

[bib7] Bellprat O , Guemas V, Doblas-Reyes F, Donat MG. 2019. Towards reliable extreme weather and climate event attribution. Nature Communications 10: 1732. 10.1038/s41467-019-09729-2 PMC6465259 30988387

[bib8] Ben-Romdhane H , Al-Musallami M, Marpu PR, Ouarda TBMJ, Ghedira H. 2018. Change detection using remote sensing in a reef environment of the UAE during the extreme event of El Niño 2015–2016. International Journal of Remote Sensing 39: 6358–6382.

[bib9] Betancourt JL. 2012. Reflections on the relevance of history in a nonstationary world. Pages 305–318 in Wiens JA, Hayward GD, Safford HD, Giffen C, eds. Historical Environmental Variation in Conservation and Natural Resource Management. Wiley.

[bib10] Blain S , et al. 2004. High frequency monitoring of the coastal marine environment using the MAREL buoy. Journal of Environmental Monitoring 6: 569–575. 15173911 10.1039/b314073c

[bib11] Boersma KS , Nickerson A, Francis CD, Siepielski AM. 2016. Climate extremes are associated with invertebrate taxonomic and functional composition in mountain lakes. Ecology and Evolution 6: 8094–8106. 27878081 10.1002/ece3.2517 PMC5108261

[bib12] Boucek RE , Rehage JS. 2014. Climate extremes drive changes in functional community structure. Global Change Biology 20: 1821–1831. 24733813 10.1111/gcb.12574

[bib13] Brantley SL , et al. 2017. Designing a network of critical zone observatories to explore the living skin of the terrestrial Earth. Earth Surface Dynamics 5: 841–860.

[bib14] Breshears DD , et al. 2005. Regional vegetation die-off in response to global-change-type drought. Proceedings of the National Academy of Sciences 102: 15144–15148. 10.1073/pnas.0505734102 PMC1250231 16217022

[bib15] Broska LH , Poganietz W-R, Vögele S. 2020. Extreme events defined: A conceptual discussion applying a complex systems approach. Futures 115: 102490.

[bib16] Burns DA , Pellerin BA, Miller MP, Capel PD, Tesoriero AJ, Duncan JM. 2019. Monitoring the riverine pulse: Applying high-frequency nitrate data to advance integrative understanding of biogeochemical and hydrological processes. WIREs. Water 6: e1348.

[bib17] Carbon Brief . 2021. Mapped: How climate change affects extreme weather around the world. Carbon Brief. www.carbonbrief.org/mapped-how-climate-change-affects-extreme-weather-around-the-world.

[bib18] Cavanaugh KC , Reed DC, Bell TW, Castorani MCN, Beas-Luna R. 2019. Spatial variability in the resistance and resilience of giant kelp in southern and Baja California to a multiyear heatwave. Frontiers in Marine Science 6: 00413.

[bib19] Chauhan P , Singh N, Chauniyal DD, Ahluwalia RS, Singhal M. 2017. Differential behaviour of a Lesser Himalayan watershed in extreme rainfall regimes. Journal of Earth System Science 126: 22.

[bib20] Chen Y-J , Nicholson E, Cheng S-T. 2020. Using machine learning to understand the implications of meteorological conditions for fish kills. Scientific Reports 10: 17003. 33046733 10.1038/s41598-020-73922-3 PMC7550581

[bib21] Cheruvelil KS , Soranno PA. 2018. Data-Intensive ecological research is catalyzed by open science and team science. BioScience 68: 813–822.

[bib22] Cheruvelil KS , Soranno PA, Weathers KC, Hanson PC, Goring SJ, Filstrup CT, Read EK. 2014. Creating and maintaining high-performing collaborative research teams: The importance of diversity and interpersonal skills. Frontiers in Ecology and the Environment 12: 31–38.

[bib23] Cottingham KL , Lennon JT, Brown BL. 2005. Knowing when to draw the line: Designing more informative ecological experiments. Frontiers in Ecology and the Environment 3: 145–152.

[bib24] Culumber ZW , Anaya-Rojas JM, Booker WW, Hooks AP, Lange EC, Pluer B, Ramírez-Bullón N, Travis J. 2019. Widespread biases in ecological and evolutionary studies. BioScience 69: 631–640.

[bib25] Curtis AN , Tiemann JS, Douglass SA, Davis MA, Larson ER. 2020. High stream flows dilute environmental DNA (eDNA) concentrations and reduce detectability. Diversity and Distributions.

[bib26] Darling ES , et al. 2019. Social-environmental drivers inform strategic management of coral reefs in the Anthropocene. Nature Ecology and Evolution 3: 1341–1350. 31406279 10.1038/s41559-019-0953-8

[bib27] Des Roches S , et al. 2021. Socio-eco-evolutionary dynamics in cities. Evolutionary Applications 14: 248–267. 33519968 10.1111/eva.13065 PMC7819562

[bib28] DiBattista JD , Reimer JD, Stat M, Masucci GD, Biondi P, De Brauwer M, Wilkinson SP, Chariton AA, Bunce M. 2020. Environmental DNA can act as a biodiversity barometer of anthropogenic pressures in coastal ecosystems. Scientific Reports 10: 8365. 32433472 10.1038/s41598-020-64858-9 PMC7239923

[bib29] Dietze MC. 2017. Ecological Forecasting. Princeton University Press.

[bib30] Dietze MC , et al. 2018. Iterative near-term ecological forecasting: Needs, opportunities, and challenges. Proceedings of the National Academy of Sciences 115: 1424–1432. 10.1073/pnas.1710231115 PMC5816139 29382745

[bib31] Di Marco M et al. 2017. Changing trends and persisting biases in three decades of conservation science. Global Ecology and Conservation 10: 32–42.

[bib32] Duarte B et al. 2018. Climate change impacts on seagrass meadows and macroalgal forests: An integrative perspective on acclimation and adaptation potential. Frontiers in Marine Science 5: 00190.

[bib33] Durden JM , Luo JY, Alexander H, Flanagan AM, Grossmann L. 2017. Integrating ‘big data’ into aquatic ecology: Challenges and opportunities. Limnology and Oceanography Bulletin 26: 101–108.

[bib34] Ensign S , Arscott D, Hicks S, Aufdenkampe A, Muenz T, Jackson J, Bressler D. 2019. A digital mayfly swarm is emerging. Eos 100: EO116611.

[bib35] Fancy SG , Gross JE, Carter SL. 2009. Monitoring the condition of natural resources in US national parks. Environmental Monitoring and Assessment 151: 161–174. 18509737 10.1007/s10661-008-0257-y

[bib36] Farley SS , Dawson A, Goring SJ, Williams JW. 2018. Situating ecology as a big-data science: Current advances, challenges, and solutions. BioScience 68: 563–576.

[bib37] Farrell KJ , Weathers KC, Sparks SH, Brentrup JA, Carey CC, Dietze MC, Foster JR, Grayson KL, Matthes JH, SanClements MD. 2021. Training macrosystems scientists requires both interpersonal and technical skills. Frontiers in Ecology and the Environment 19: 39–46.

[bib38] Field CB , Barros V, Stocker TF, Dahe Q. 2012. Glossary of terms. Pages 555–564 in Field CB et al., eds. Managing the Risks of Extreme Events and Disasters to Advance Climate Change Adaptation: Special Report of the Intergovernmental Panel on Climate Change. Cambridge University Press.

[bib39] Frölicher TL , Fischer EM, Gruber N. 2018. Marine heatwaves under global warming. Nature 560: 360–364. 30111788 10.1038/s41586-018-0383-9

[bib40] Gaiser EE , et al. 2020. Long-term ecological research and evolving frameworks of disturbance ecology. BioScience 70: 141–156.

[bib41] Gochis D , et al. 2015. The great Colorado flood of September 2013. Bulletin of the American Meteorological Society 96: 1461–1487.

[bib42] Godsey SE , Hartmann J, Kirchner JW. 2019. Catchment chemostasis revisited: Water quality responds differently to variations in weather and climate. Hydrological Processes 33: 3056–3069.

[bib43] Greig-Smith P , Cragg JB. 1964. Advances in ecological research. Journal of Ecology 52: 795.

[bib44] Grimm NB , Grove JG, Pickett STA, Redman CL. 2000. Integrated approaches to long-term studies of urban ecological systems: Urban ecological systems present multiple challenges to ecologists: Pervasive human impact and extreme heterogeneity of cities, and the need to integrate social and ecological approaches, concepts, and theory. BioScience 50: 571–584.

[bib45] Grimm NB , Pickett STA, Hale RL, Cadenasso ML. 2017. Does the ecological concept of disturbance have utility in urban social–ecological–technological systems? Ecosystem Health and Sustainability 3: e01255.

[bib46] Grossmann MM , Gallager SM, Mitarai S. 2015. Continuous monitoring of near-bottom mesoplankton communities in the East China Sea during a series of typhoons. Journal of Oceanography 71: 115–124.

[bib47] Gruber N , Boyd PW, Frölicher TL, Vogt M. 2021. Biogeochemical extremes and compound events in the ocean. Nature 600: 395–407. 34912083 10.1038/s41586-021-03981-7

[bib48] Guillou N , Chapalain G. 2021. Machine learning methods applied to sea level predictions in the upper part of a tidal estuary. Oceanologia 63: 531–544.

[bib49] Guswa AJ , et al. 2020. Advancing ecohydrology in the 21st century: A convergence of opportunities. Ecohydrology 13: e2208.

[bib50] Haberl H , et al. 2006. From LTER to LTSER: Conceptualizing the socioeconomic dimension of long-term socioecological research. Ecology and Society 11: 13.

[bib51] Hampton SE , Strasser CA, Tewksbury JJ, Gram WK, Budden AE, Batcheller AL, Duke CS, Porter JH. 2013. Big data and the future of ecology. Frontiers in Ecology and the Environment 11: 156–162.

[bib52] Hamshaw SD , Dewoolkar MM, Schroth AW, Wemple BC, Rizzo DM. 2018. A new machine-learning approach for classifying hysteresis in suspended-sediment discharge relationships using high-frequency monitoring data. Water Resources Research 54: 4040–4058.

[bib53] Hartmann J , Lauerwald R, Moosdorf N. 2014. A brief overview of the GLObal RIver chemistry database, GLORICH. Procedia Earth and Planetary Science 10: 23–27.

[bib54] Heffernan JB , et al. 2014. Macrosystems ecology: Understanding ecological patterns and processes at continental scales. Frontiers in Ecology and the Environment 12: 5–14.

[bib55] Herring SC , Hoerling MP, Peterson TC, Stott PA. 2014. Explaining extreme events of 2013 from a climate perspective. Bulletin of the American Meteorological Society 95: S1–S104.

[bib56] Hobday AJ , et al. 2016. A hierarchical approach to defining marine heatwaves. Progress in Oceanography 141: 227–238.

[bib57] Hobday A , et al. 2018. Categorizing and naming marine heatwaves. Oceanography 31: 162–173.

[bib58] Huang T-Y , Downs MR, Ma J, Zhao B. 2020. Collaboration across time and space in the LTER Network. BioScience 70: 353–364.

[bib59] Javed A , Hamshaw SD, Lee BS, Rizzo DM. 2021. Multivariate event time series analysis using hydrological and suspended sediment data. Journal of Hydrology 593: 125802.

[bib60] Johnson JC , Christian RR, Brunt JW, Hickman CR, Waide RB. 2010. Evolution of collaboration within the US Long Term Ecological Research Network. BioScience 60: 931–940.

[bib61] Jones KR , Watson JEM, Possingham HP, Klein CJ. 2016. Incorporating climate change into spatial conservation prioritisation: A review. Biological Conservation 194: 121–130.

[bib62] Kendrick GA , et al. 2019. A systematic review of how multiple stressors from an extreme event drove ecosystem-wide loss of resilience in an iconic seagrass community. Frontiers in Marine Science 6: 00455.

[bib63] Klug JL , et al. 2012. Ecosystem effects of a tropical cyclone on a network of lakes in northeastern North America. Environmental Science and Technology 46: 11693–11701. 23016881 10.1021/es302063v

[bib64] Kohler LE , Silverstein J, Rajagopalan B. 2020. Resilience of on-site wastewater treatment systems after extreme storm event. Journal of Sustainable Water in the Built Environment 6: 04020008.

[bib65] Krause S , Lewandowski J, Dahm CN, Tockner K. 2015. Frontiers in real-time ecohydrology: A paradigm shift in understanding complex environmental systems. Ecohydrology 8: 529–537.

[bib66] Kreyling J , Jentsch A, Beier C. 2014. Beyond realism in climate change experiments: Gradient approaches identify thresholds and tipping points. Ecology Letters 17: 125–e1. 10.1111/ele.12193 24341985

[bib67] LaDeau SL , Han BA, Rosi-Marshall EJ, Weathers KC. 2017. The next decade of big data in ecosystem science. Ecosystems 20: 274–283.

[bib68] Larson EI , Poff NL, Atkinson CL, Flecker AS. 2018. Extreme flooding decreases stream consumer autochthony by increasing detrital resource availability. Freshwater Biology 63: 1483–1497.

[bib69] Leigh C , Bush A, Harrison ET, Ho SS, Luke L, Rolls RJ, Ledger ME. 2015. Ecological effects of extreme climatic events on riverine ecosystems: Insights from Australia. Freshwater Biology 60: 2620–2638.

[bib70] Li L , Chakraborty P. 2020. Slower decay of landfalling hurricanes in a warming world. Nature 587: 230–234. 33177666 10.1038/s41586-020-2867-7

[bib71] Li M , Zhang F, Barnes S, Wang X. 2020. Assessing storm surge impacts on coastal inundation due to climate change: Case studies of Baltimore and Dorchester County in Maryland. Natural Hazards 103: 2561–2588.

[bib72] Lohner TW , Dixon DA. 2013. The value of long-term environmental monitoring programs: An Ohio River case study. Environmental Monitoring and Assessment 185: 9385–9396. 23715733 10.1007/s10661-013-3258-4 PMC3787800

[bib73] Mächler E , Salyani A, Walser J-C, Larsen A, Schaefli B, Altermatt F, Ceperley N. 2021. Environmental DNA simultaneously informs hydrological and biodiversity characterization of an Alpine catchment. Hydrology and Earth System Sciences 25: 735–753.

[bib74] Mao F , Khamis K, Krause S, Clark J, Hannah DM. 2019. Low-cost environmental sensor networks: Recent advances and future directions. Frontiers of Earth Science 7: 00221.

[bib75] Maxwell SL , Butt N, Maron M, McAlpine CA, Chapman S, Ullmann A, Segan DB, Watson JEM. 2019. Conservation implications of ecological responses to extreme weather and climate events. Diversity and Distributions 25: 613–625.

[bib76] Mcleman R , Smit B. 2006. Vulnerability to climate change hazards and risks: Crop and flood insurance. The Canadian geographer. Geographe Canadien 50: 217–226.

[bib77] McPhillips LE , et al. 2018. Defining extreme events: A cross-disciplinary review. Earth's Future 6: 441–455.

[bib78] Meierdiercks KL , Smith JA, Baeck ML, Miller AJ. 2010. Analyses of urban drainage network structure and its impact on hydrologic response1. Journal of the American Water Resources Association 46: 932–943.

[bib79] Milly PCD , Wetherald RT, Dunne KA, Delworth TL. 2002. Increasing risk of great floods in a changing climate. Nature 415: 514–517. 11823857 10.1038/415514a

[bib80] Nielsen UN , Wall DH, Adams BJ, Virginia RA, Ball BA, Gooseff MN, McKnight DM. 2012. The ecology of pulse events: Insights from an extreme climatic event in a polar desert ecosystem. Ecosphere 3: 17.

[bib81] Njue N , Stenfert Kroese J, Gräf J, Jacobs SR, Weeser B, Breuer L, Rufino MC. 2019. Citizen science in hydrological monitoring and ecosystem services management: State of the art and future prospects. Science of the Total Environment 693: 133531. 31635016 10.1016/j.scitotenv.2019.07.337

[bib82] Ntelekos AA , Smith JA, Baeck ML, Krajewski WF, Miller AJ, Goska R. 2008. Extreme hydrometeorological events and the urban environment: Dissecting the 7 July 2004 thunderstorm over the Baltimore, MD, metropolitan region. Water Resources Research 44: WR006346.

[bib83] Osterback A-MK , Kern CH, Kanawi EA, Perez JM, Kiernan JD. 2018. The effects of early sandbar formation on the abundance and ecology of Coho salmon (*Oncorhynchus kisutch*) and steelhead trout (*Oncorhynchus mykiss*) in a central California coastal lagoon. Canadian Journal of Fisheries and Aquatic Sciences/Journal Canadien des Sciences Halieutiques et Aquatiques 75: 2184–2197.

[bib84] Paerl HW , Crosswell JR, Van Dam B, Hall NS, Rossignol KL, Osburn CL, Hounshell AG, Sloup RS, Harding LW. 2018. Two decades of tropical cyclone impacts on North Carolina's estuarine carbon, nutrient and phytoplankton dynamics: Implications for biogeochemical cycling and water quality in a stormier world. Biogeochemistry 141: 307–332.

[bib85] Paerl HW , Hall NS, Hounshell AG, Luettich RA Jr, Rossignol KL, Osburn CL, Bales J. 2019. Recent increase in catastrophic tropical cyclone flooding in coastal North Carolina, USA: Long-term observations suggest a regime shift. Scientific Reports 9: 10620. 31337803 10.1038/s41598-019-46928-9 PMC6650462

[bib86] Palmer WC. 1965. Meteorological Drought. US Department of Commerce, Weather Bureau.

[bib87] Patrick CJ , et al. 2020. A system level analysis of coastal ecosystem responses to hurricane impacts. Estuaries and Coasts 43: 943–959.

[bib88] Pennington DD , Simpson GL, McConnell MS, Fair JM, Baker RJ. 2013. Transdisciplinary research, transformative learning, and transformative science. BioScience 63: 564–573.

[bib89] Peters DPC , Loescher HW, SanClements MD, Haversustad KM. 2014. Taking the pulse of a continent: Expanding site-based research infrastructure for regional- to continental-scale ecology. Ecosphere 5: 29.

[bib90] Poff NL , Larson EI, Salerno PE, Morton SG, Kondratieff BC, Flecker AS, Zamudio KR, Funk WC. 2018. Extreme streams: Species persistence and genomic change in montane insect populations across a flooding gradient. Ecology Letters 21: 525–535. 29430810 10.1111/ele.12918

[bib91] Politikos DV , Petasis G, Katselis G. 2021. Interpretable machine learning to forecast hypoxia in a lagoon. Ecological Informatics 66: 101480.

[bib92] Rammer W , Seidl R. 2019. Harnessing deep learning in ecology: An example predicting bark beetle outbreaks. Frontiers in Plant Science 10: 1327. 31719829 10.3389/fpls.2019.01327 PMC6827389

[bib93] Raymond C , et al. 2020. Understanding and managing connected extreme events. Nature Climate Change 10: 611–621.

[bib94] Redmond MD , Law DJ, Field JP, Meneses N, Carroll CJW, Wion AP, Breshears DD, Cobb NS, Dietze MC, Gallery RE. 2019. Targeting extreme events: Complementing near-term ecological forecasting with rapid experiments and regional surveys. Frontiers of Environmental Science and Engineering in China 7: 183.

[bib95] Reisinger AJ , Rosi EJ, Bechtold HA, Doody TR, Kaushal SS, Groffman PM. 2017. Recovery and resilience of urban stream metabolism following Superstorm Sandy and other floods. Ecosphere 8: e01776.

[bib96] Reyers B , Roux DJ, O'farrell PJ. 2010. Can ecosystem services lead ecology on a transdisciplinary pathway? Environmental Conservation 37: 501–511.

[bib97] Rojas R , Feyen L, Watkiss P. 2013. Climate change and river floods in the European Union: Socio-economic consequences and the costs and benefits of adaptation. Global Environmental Change: Human and Policy Dimensions 23: 1737–1751.

[bib98] Rüegg J , Conn CC, Anderson EP, Battin TJ, Bernhardt ES, Boix Canadell M, Bonjour SM, Hosen JD, Marzolf NS, Yackulic CB. 2021. Thinking like a consumer: Linking aquatic basal metabolism and consumer dynamics. Limnology and Oceanography Letters 6: 1–17.

[bib99] Salt GW. 1979. A comment on the use of the term emergent properties. American Naturalist 113: 145–148.

[bib100] Seneviratne SI , et al. 2012. Changes in climate extremes and their impacts on the natural physical environment. Pages 109–230 in Field CB et al., eds. Managing the Risks of Extreme Events and Disasters to Advance Climate Change Adaptation: Special Report of the Intergovernmental Panel on Climate Change. Cambridge University Press.

[bib101] Setunge S , Lokuge W, Mohseni H, Karunasena W. 2014. Vulnerability of road bridge infrastructure under extreme flood events. Paper presented at the AFAC and Bushfire and Natural Hazards CRC Conference, 5 September 2014, in Wellington, New Zealand.

[bib102] Slater L , Villarini G, Archfield S, Faulkner D, Lamb R, Khouakhi A, Yin J. 2021. Global changes in 20-year, 50-year, and 100-year river floods. Geophysical Research Letters 48: e2020GL091824.

[bib103] Slette IJ , Post AK, Awad M, Even T, Punzalan A, Williams S, Smith MD, Knapp AK. 2019. How ecologists define drought, and why we should do better. Global Change Biology 25: 3193–3200. 31276260 10.1111/gcb.14747

[bib104] Smale DA , et al. 2019. Marine heatwaves threaten global biodiversity and the provision of ecosystem services. Nature Climate Change 9: 306–312.

[bib105] Stockwell JD , et al. 2020. Storm impacts on phytoplankton community dynamics in lakes. Global Change Biology 26: 2756–2784. 32133744 10.1111/gcb.15033 PMC7216882

[bib106] Tahsin S , Medeiros SC, Singh A. 2018. Assessing the resilience of coastal wetlands to extreme hydrologic events using vegetation indices: A review. Remote Sensing 10: 1390.

[bib107] Tamminga AD , Eaton BC, Hugenholtz CH. 2015. UAS-based remote sensing of fluvial change following an extreme flood event. Earth Surface Processes and Landforms 40: 1464–1476.

[bib108] Tauro F , et al. 2018. Measurements and observations in the XXI century (MOXXI): Innovation and multi-disciplinarity to sense the hydrological cycle. Hydrological Sciences Journal 63: 169–196.

[bib109] Thomsen PF , Willerslev E. 2015. Environmental DNA: An emerging tool in conservation for monitoring past and present biodiversity. Biological Conservation 183: 4–18.

[bib110] Tonkin JD , Merritt DM, Olden JD, Reynolds LV, Lytle DA. 2018. Flow regime alteration degrades ecological networks in riparian ecosystems. Nature Ecology and Evolution 2: 86–93. 29180707 10.1038/s41559-017-0379-0

[bib111] Trigg MA , Michaelides K, Neal JC, Bates PD. 2013. Surface water connectivity dynamics of a large scale extreme flood. Journal of Hydrology 505: 138–149.

[bib112] Tydecks L , Bremerich V, Jentschke I, Likens GE, Tockner K. 2016. Biological field stations: A global infrastructure for research, education, and public engagement. BioScience 66: 164–171.

[bib113] [UNDRR] United Nations Office for Disaster Risk Reduction . 2020. The Human Cost of Disasters: An Overview of the Last 20 Years (2000–2019). UNDRR. https://dds.cepal.org/redesoc/publication?id=5361.

[bib114] van de Pol M , Jenouvrier S, Cornelissen JHC, Visser ME. 2017. Behavioural, ecological and evolutionary responses to extreme climatic events: Challenges and directions. Philosophical Transactions of the Royal Society B 372: 0134. 10.1098/rstb.2016.0134 PMC5434086 28483865

[bib115] Vörösmarty CJ , et al. 2010. Global threats to human water security and river biodiversity. Nature 467: 555–561. 20882010 10.1038/nature09440

[bib116] Wang K , Franklin SE, Guo X, Cattet M. 2010. Remote sensing of ecology, biodiversity and conservation: A review from the perspective of remote sensing specialists. Sensors 10: 9647–9667. 22163432 10.3390/s101109647 PMC3231003

[bib117] Weathers KC , et al. 2016. Frontiers in ecosystem ecology from a community perspective: The future is boundless and bright. Ecosystems 19: 753–770.

[bib118] Woolway RI , Jennings E, Shatwell T, Golub M, Pierson DC, Maberly SC. 2021a. Lake heatwaves under climate change. Nature 589: 402–407. 33473224 10.1038/s41586-020-03119-1

[bib119] Woolway RI , Kraemer BM, Zscheischler J, Albergel C. 2021b. Compound hot temperature and high chlorophyll extreme events in global lakes. Environmental Research Letters 16: 124066.

[bib120] Wright Morton L , Eigenbrode S, Martin T. 2015. Architectures of adaptive integration in large collaborative projects. Ecology and Society 20: 5.

[bib121] Yin J , Gentine P, Zhou S, Sullivan SC, Wang R, Zhang Y, Guo S. 2018. Large increase in global storm runoff extremes driven by climate and anthropogenic changes. Nature Communications 9: 4389. 10.1038/s41467-018-06765-2 PMC6197252 30348951

[bib122] Zhang J , Sun F, Lai W, Lim WH, Liu W, Wang T, Wang P. 2019. Attributing changes in future extreme droughts based on PDSI in china. Journal of Hydrology 573: 607–615.

[bib123] Zscheischler J , et al. 2018. Future climate risk from compound events. Nature Climate Change 8: 469–477.

