# Supplementary material for: Preparing Aquatic Research for an Extreme Future: Call for Improved Definitions and Responsive, Multidisciplinary Approaches
Source: Bioscience. 2022 May 4;72(6):508–20. doi: 10.1093/biosci/biac020 (PMC9169894; doi:10.1093/biosci/biac020)
Supplement: biac020_Supplemental_File [file biac020_supplemental_file.docx]

Supplementary Material for

**Preparing aquatic research for an extreme future: Call for improved definitions and responsive, multidisciplinary approaches**

**Authors**

Lillian R. Aoki^1^^, Margaret Mars Brisbin^2^, Alexandria G. Hounshell^3,#^, Dustin W. Kincaid^4^, Erin Larson^5^, Brandon J, Samson^6,*^, Arial J. Shogren^7,†^, Rachel S. Smith^8^, Jenna Sullivan-Stack^9^

**Correspondence to:** Lillian Aoki, [lra53@cornell.edu](mailto:lra53@cornell.edu)

This file includes Supplementary Table S1 and Supplementary Figures S1-S5

**Table S1**. Search terms used to identify relevant literature targeting three categories of aquatic ecosystems (freshwater, coastal, and marine) and four types of extreme events (heatwaves, storms, floods, and drought). The literature search was conducted as follows: (general search terms separated by OR) AND (ecosystem search terms separated by OR) AND (event search terms separated by OR). See main text for other details of the literature search.

| **General search terms** | **Ecosystem search terms** | **Event search terms** |
| --- | --- | --- |
| extreme event  ecology | aquatic  freshwater  stream  lake  river  creek  wetland  coast*  estruar*  *tidal  marine  ocean | heat*  temperature  warm*  storm  wind  hurricane  typhoon  cyclone  flood  precipitation  drought  desiccation |

**
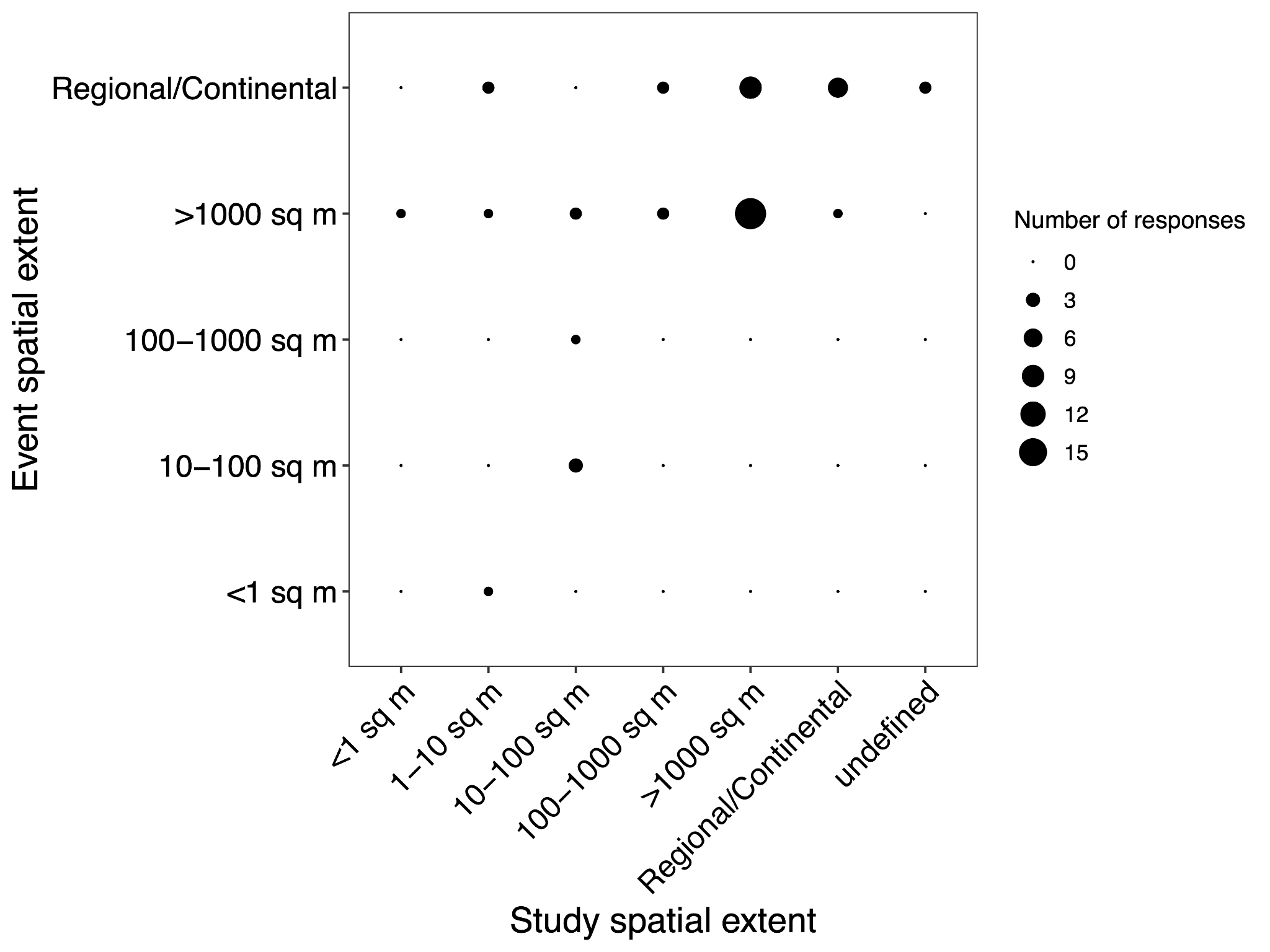
**

**Fig. S1:** Number of responses from literature review comparing the spatial extent of the study to the spatial extent of the extreme event across different spatial scales (from < 1 sq m to Regional/Continental). The number of responses is indicated by circle size, which is scaled by area.

**
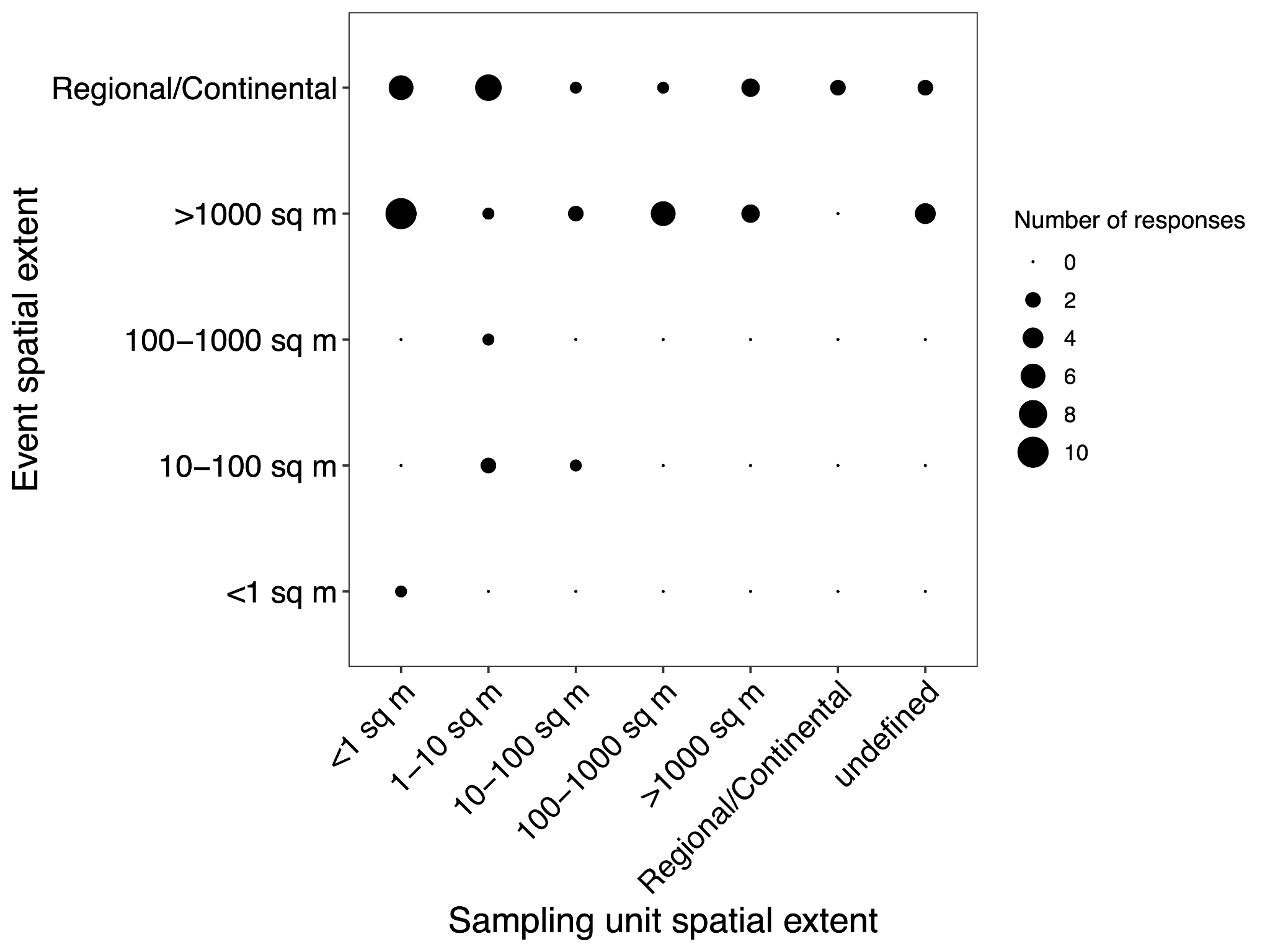
**

**Fig. S2:** Number of responses from the literature review comparing the sampling unit spatial extent and the extreme event spatial extent across different spatial scales (from < 1 sq m to Regional/Continental). The number of responses is indicated by circle size, which is scaled by area.

**
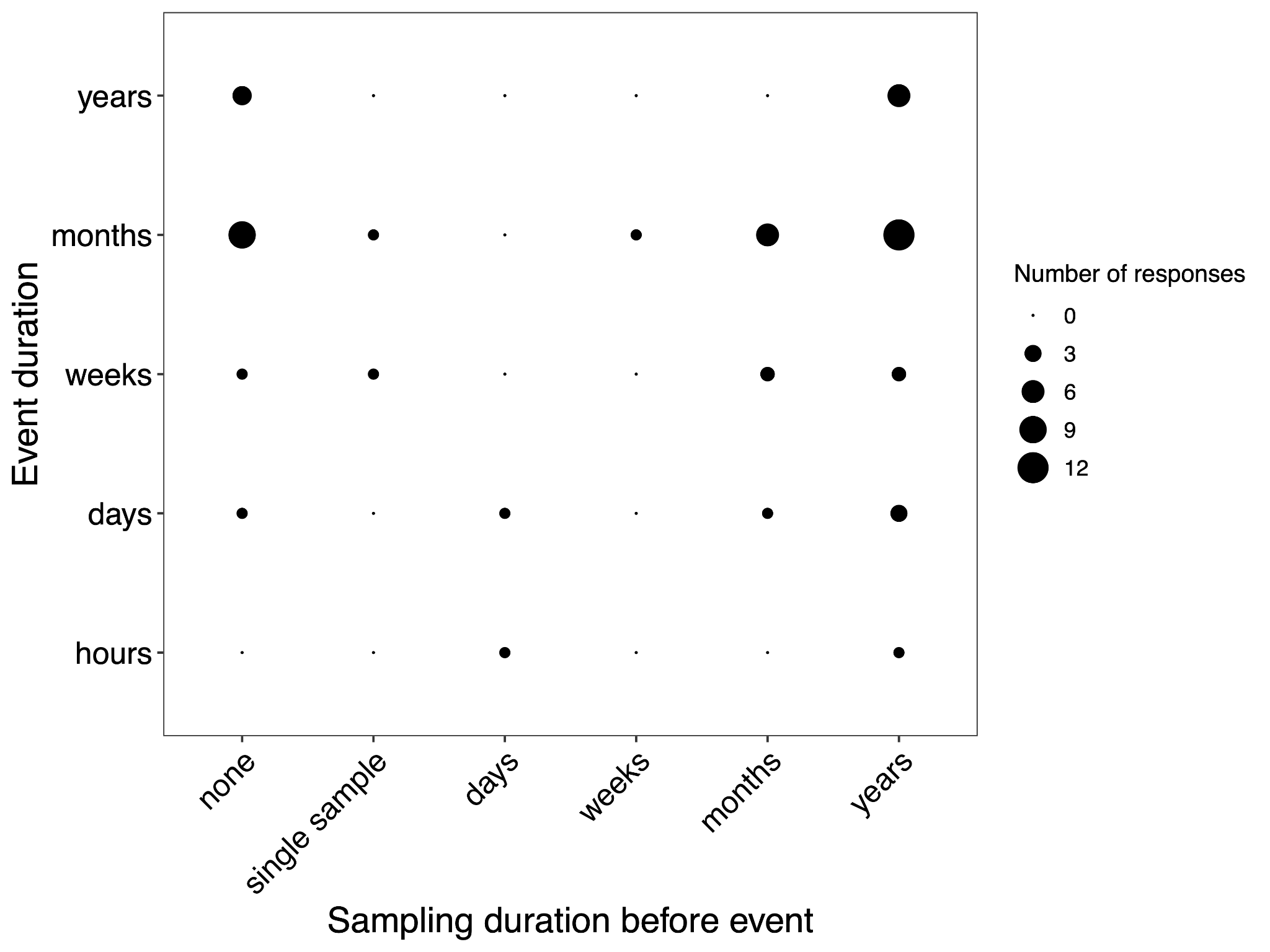
**

**Fig. S3:** Number of responses from literature review comparing the sampling duration before an extreme event and the event duration across different temporal scales (from no sampling to years). The number of responses is indicated by circle size, which is scaled by area.

**
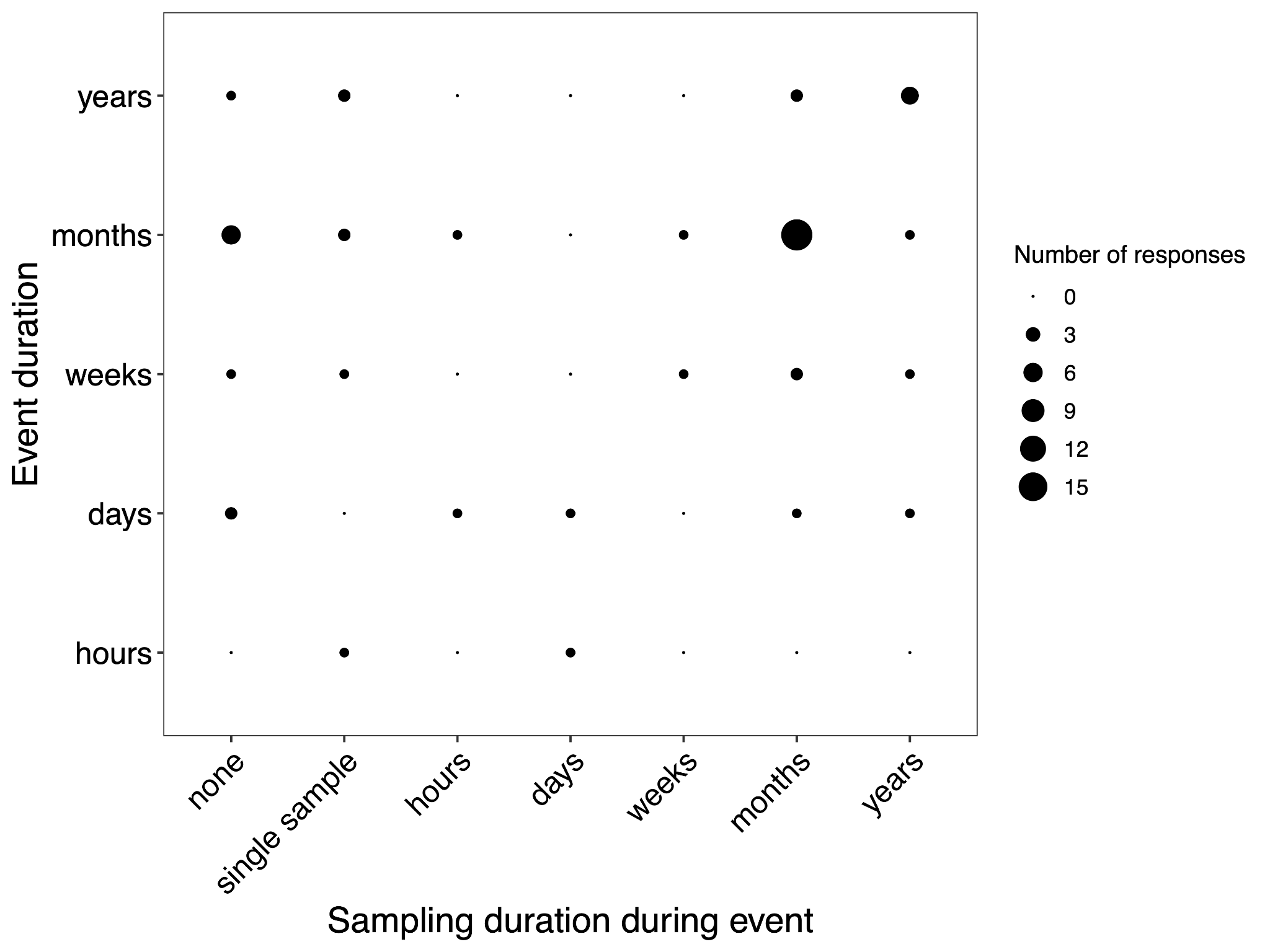
**

**Fig. S4:** Number of responses from literature review comparing the sampling duration during an extreme event and the event duration across different temporal scales (from no sampling to years). The number of responses is indicated by circle size, which is scaled by area.

**
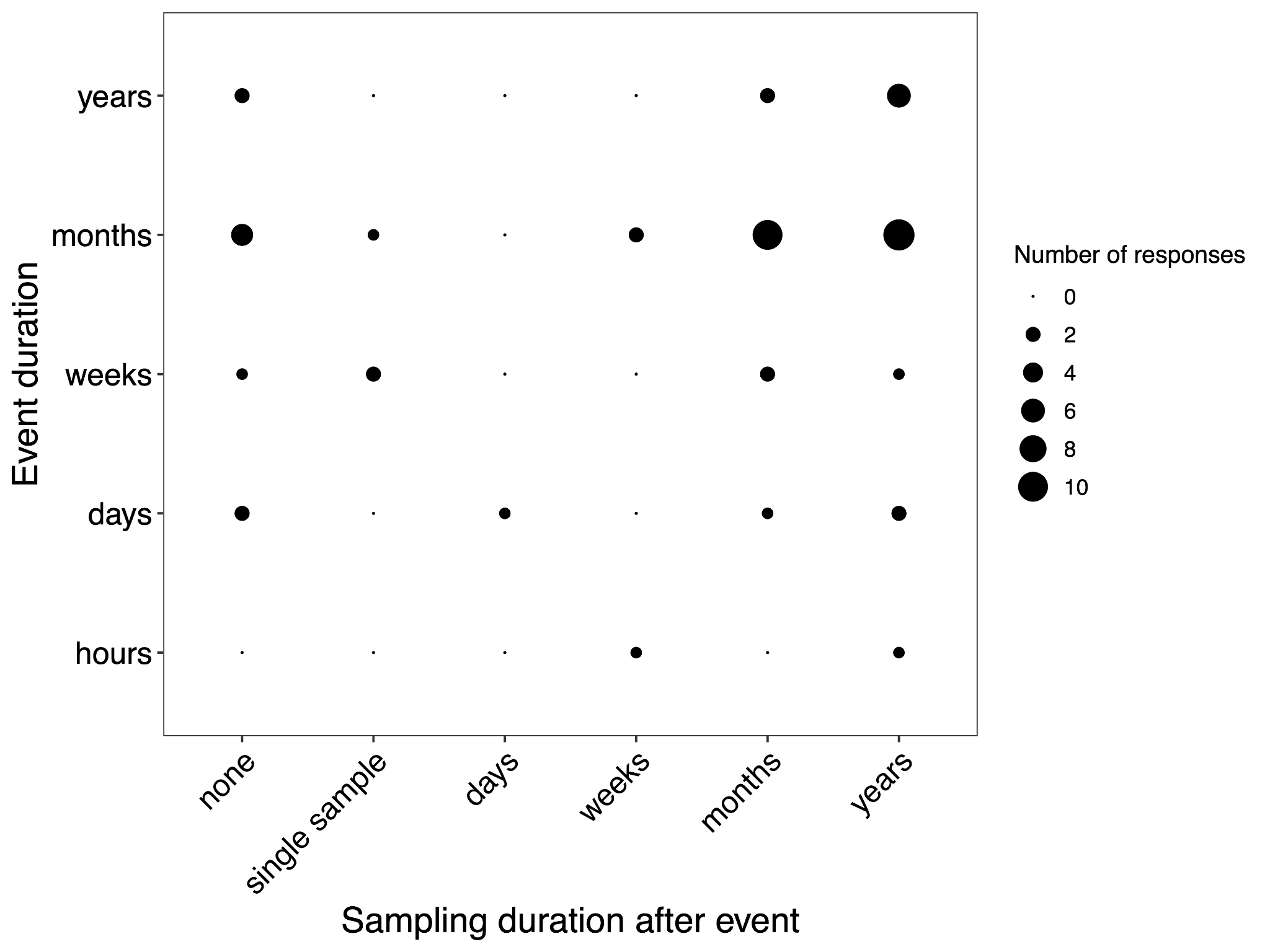
**

**Fig. S5:** Number of responses from literature review comparing the sampling duration after an extreme event and the event duration across different temporal scales (from no sampling to years). The number of responses is indicated by circle size, which is scaled by area.
